# Identifying palliative care needs of patients with non-communicable diseases in Indonesia using the SPICT tool: a descriptive cross-sectional study

**DOI:** 10.1186/s12904-021-00881-5

**Published:** 2022-01-24

**Authors:** Christantie Effendy, Jony Francisco Dos Santos Silva, Retna Siwi Padmawati

**Affiliations:** 1 Department of Medical Surgical Nursing, Faculty of Medicine, Public Health and Nursing, Universitas Gadjah Mada, Jl. Farmako, Sekip Utara, Depok District, Sleman, Special Region of Yogyakarta, Yogyakarta, 55281 Indonesia; 2 Center for Bioethics and Medical Humanities, Faculty of Medicine, Public Health, and Nursing, Universitas Gadjah Mada, Yogyakarta, Indonesia; 3 Hospital Nacional Guido Valadares, Dili, Timor-Leste; 4 Department of Health Behavior, Environment, and Social Medicine; Faculty of Medicine, Public Health, and Nursing, Universitas Gadjah Mada, Yogyakarta, Indonesia; 5 Graduate Study Program on Bioethics, Universitas Gadjah Mada, Yogyakarta, Indonesia

**Keywords:** Palliative care, Non-communicable diseases, Screening, SPICT tool, Indonesia

## Abstract

**Background:**

In Indonesia, Non-Communicable Diseases (NCD) are a contributing factor to mortality with most cases involving heart disease, cancer, chronic lung disease and diabetes. Accordingly, the identification of palliative care needs is very important as a first step in providing palliative care for these patients with NCD. However, currently there is no national standardized tool nor guidance system for identifying palliative care needs of NCD patients in Indonesia. The Supportive and Palliative Care Indicators Tool (SPICT) has been used worldwide for screening palliative care needs. This study aimed to identify palliative care needs in NCD patients using the SPICT tool.

**Methods:**

This descriptive study used a cross-sectional design. Sampling technique used convenience sampling with a total sample of 124 adult patients with NCD in Dr. Sardjito Hospital Yogyakarta. Data collection used the Indonesian version of the SPICT. Data analyses used descriptive statistics and chi-square tests with *p* < 0,05 set as significant. Additionally, the prevalence of patients requiring palliative care was also calculated.

**Results:**

The patients with NCD requiring palliative care who were screened using the SPICT tool were 61.3%. The nurses identified only 17.7%, while the physicians identified only 9.7%. The overall agreement of the clinician’s assessments to the researchers was < 32%. Meanwhile, agreement with nurses was 31 and 25% with the physicians.

**Conclusions:**

These results highlight that by using the SPICT tool, recognition of hospitalized patients with NCD needing palliative care increased from 10 to 18% to > 61%. The Indonesian version of the SPICT tool can help the clinicians to reach meet agreement in identifying hospitalized patients who need palliative care as the first step in addressing palliative interventions for patients with NCD. It can provide several benefits in screening patients with NCD from the beginning of diagnosis.

## Background

Non-Communicable Diseases (NCD) are the main cause of death in several countries. The highest mortality of NCD is caused by cardiovascular disease (CVD), cancer, chronic lung disease, and diabetes, followed by other chronic diseases [1]. This trend includes Indonesia, which is experiencing a high burden of NCDs [2]. Although efforts to prevent and control NCDs have increased nationally, the risk factors and morbidity of NCDs show a tendency for patients with NCD to deteriorate toward end-stage diseases [2–5]. This pathogenesis can result in progressive declining of functional systems with the accompanying physical, mental and social disorders [6].

Palliative care is recognized as a fundamental human right and an ethical obligation among care providers [7]. Palliative care has an important role to play in supporting caregivers and families of patients with chronic disease, such as those with NCDs [8], particularly ones who cannot be treated medically [9]. Generally, palliative care can help families and patients to reach their needs holistically [10]. The goal of palliative care is to relieve the suffering of patients and their families by the comprehensive assessment and treatment of physical, psychosocial, and spiritual symptoms experienced by patients [11, 12]. Providing palliative care to patients with NCD begins by identifying the patient’s need for palliative care. It is very important as a first step in providing palliative care to patients with NCD [13]. Palliative care will help to improve their quality of life and ensure a calm, peaceful and dignified death [10, 13, 14]. Delays in identifying patients’ palliative care needs will have a negative impact on their quality of life [14].

There is possibility of disagreement between clinicians in identifying palliative care needs of the patient with NCD [15, 16]. If the clinicians consider that the patient does not need palliative care, even though they do, then this dilemma will lead to a condition where the patient does not receive the necessary palliative care that is a protected human right [7]. Therefore, it is very important to have a measuring tool that can assist clinicians in making decisions to determine whether patients need palliative care or not [17].

The Supportive & Palliative Care Indicators Tool (SPICT) is an instrument used worldwide for palliative care screening. The advantages of SPICT are having general and clinical indicators that can assess the severity of disease which can be easily used by health workers. The instrument assists the clinical considerations of multidisciplinary teams in identifying patients with deteriorating health who need palliative care in a hospital [18].

Data on patients with palliative care needs are not comprehensively available yet in Indonesia [19], particularly in Yogyakarta. So far, there are no national standards nor any registration system for identifying the needs of palliative patients [19]. It is important to establish an instrument as a gold standard for the accurate screening of palliative care needs [20]. In this study we used the SPICT as a gold standard instrument as compared to clinicians (physicians and nurses) judgement. More specifically, this study aimed to identify palliative care needs in NCD patients using the SPICT tool.

## Method

### Design

This descriptive, cross-sectional survey aimed to identify patients with NCD who need palliative care. Data were collected between 28 September − 15 December 2019 at Dr. Sardjito Hospital, Special Region of Yogyakarta (Yogyakarta Province) Indonesia.

### Sampling

The respondents in this study were adult patients with NCD who were hospitalized at the in-patient ward at Dr. Sardjito Hospital, Yogyakarta, Indonesia. The sampling technique was using convenience sampling. The inclusion criteria for the patients were as follows: 1) patients were diagnosed with NCD based on medical records (i.e., advanced cancer, stroke, chronic obstructive pulmonary disease (COPD), diabetes, CVD, and/or chronic kidney disease (CKD) with no minimum duration of the disease), and 2) agreed to participate in the study by signing an informed consent form. Meanwhile, the exclusion criteria were as follows: 1) patients who experienced communication barriers (installed medical devices such tracheotomy, installed high-pressure oxygen, post-oral and throat surgical treatment), and 2) re-admitted patients who have provided previous data. One hundred and fifty adult patients with NCD who were treated at Dr. Sardjito Hospital Yogyakarta were invited to participate in this study. One hundred and twenty-four (82.66%) patients agreed to participate.

### Data collection

The admitted patient was examined for the main diagnosis of NCD to be selected as an eligible respondent. With the help of a head nurse, we identified the patients who met the criteria. The head nurse introduced the researchers to the patients (and their family) and they explained in more detail about the purpose of the study. Furthermore, the researchers asked for their voluntary consent to be involved in the study by signing the informed consent form. The researchers used the SPICT tool to measure the need of palliative care of each respondent. The SPICT was completed based on the respondent’s condition and also referred to the medical record. Along with SPICT instrument, we asked the clinicians (physicians and nurses) whether those patients need palliative care.

### Measurement

The Supportive and Palliative Care Indicators Tool (SPICT™) was originally developed in 2010 by the Primary Palliative Care Research Group of the University of Edinburgh to help multidisciplinary teams to identify patients at risk of deteriorating and dying in all care settings [18]. It consists of a set of clinical indicators and has a three-part structure. The first section consists of general clinical indicators, the second details disease-specific indicators and the third provides important recommendations for reviewing and care planning [17, 18]. Patients who have been identified as requiring palliative care through SPICT™ usually have at least two common indicators [17]. The tool has been commonly used in European countries, and for this study we were permitted to translate and use it. The SPICT tool has two indicators that can be used to assess general health problems and patient limitations. The advantage of this instrument is that it can be used to assess all cases of chronic disease requiring palliative care, compared to other instruments used for specific disease cases only [18]. In this study, the English version of SPICT has been translated into the Indonesian language, then back translated into English (all English and medical terminologies were matched and met the meaning of the original version as results of the forward-back translation), and had been tested for validity and reliability. We used coefficient of reproducibility validity (Kr) and the coefficient of scalability validity (Ks) to measure the validity of the SPICT tool. The validity analysis obtained Kr = 0.99 (standard Kr > 0.90) and Ks = 0.7 (standard Ks > 0.6) using. The reliability test used the Kuder-Richardson (KR) scale, and obtained KR = 0.97 (standard > 0.60). These results indicated that the Indonesian version of SPICT is valid and reliable for use in screening patients who need palliative care.

### Data analysis

Descriptive analysis was used to analyze demographic data using frequency, mean and standard deviation (SD), with percentages. The need for palliative care was analyzed by frequency and percentages. Chi square tests with Fisher Kolmogorov Smirnov analysis was used to determine whether there were significant differences between demographic characteristics related to the need for palliative care. A value of *p* < 0.05 was considered to be statistically significant. The independent variables (gender, age, type of illness and length of illness) were selected with consideration of the need for palliative care in Indonesia, focusing on women with cancer, which was the most experienced disease by patients aged over 60 years. Duration of illness (> 6 months) was selected for consideration of the long-term care category. All of the statistical analyses were performed using the software program SPSS—version 22 (IBM Corp., Armonk, NY, USA).

## Results

### Demographic characteristics

The demographic characteristics of respondents are shown in Table 1. The majority of respondents were aged 56-65 years (33.9%) with an average age of 53 years. More than half of the respondents were female patients (58.1%), have elementary school education (38.7%), and were married (95.2%). Most of the respondents live in the DIY or Special Region of Yogyakarta (58.9%) and work as housewives (33.9%).

**Table 1:** Characteristic demographic of respondents (*n* = 124) [35]

Characteristic	n (%)
Sex	
Male	52 (41.9%)
Female	72 (58.1%)
Age (Mean ± SD)	53 ± 12.5
≤ 30 years	10 (8.1%)
31-45 years	17 (13.7%)
46-55 years	40 (32.3%)
56-65 years	42 (33.9%)
> 65 years	15 (12.0%)
Level of education	
Uneducated	3 (2.4%)
Elementary school	48 (38.7%)
Junior high school	22 (17.7%)
Senior high school	43 (34.7%)
College/University	8 (6.4%)
Marital status	
Unmarried	6 (4.8%)
Married	118 (95.2%
Residence	
Special Region of Yogyakarta	73 (58.9%)
Outside Special Region of Yogyakarta	51 (41.1%)
Occupation	
Housewives	42 (33.9%)
Farmer	23 (18.5%)
Trader	16 (12.9%)
Labourer	21 (16.9%)
Public employment	5 (4.0%)
Private sector	10 (8.1%)
Others	7 (5.7%)

(Source: Primary data, 2019)

### History of disease and medication

Table 2 shows the history of diseases and medications of respondents. The majority of the respondents suffered from cancer (71.8%) with duration of the treatment mostly less than 1 week (74.2%). Most of the length of illness since the main diagnosis was established more than 6 months (86.3%) and the frequency of hospitalizations since diagnosis was more than 2 times (99.2%).

**Table 2:** History of disease and clinical characteristic of respondents (*n* = 124)

Characteristic	n (%)
Type of diseases	
Advanced Cancer	81 (71.8%)
Cardiovascular disease	9 (7.3%)
Respiratory disease	9 (7,3%)
Liver disease	12 (9.7%)
Others (renal, neurologic, diabetes)	5 (4%)
Duration of hospitalization	
< 3 days	11 (8.9%)
3- 5 days	81 (65.3%)
6 - 7 days	9 (7.3%)
8 - 14 days	15 (12.1%)
15 - 30 days	5 (4.0%)
> 31 days	3 (2.4%)
Length of illness since diagnosed	
< 6 months	17 (13.7%)
6-12 months	48 (38.7%)
> 12 months	59 (47.6%)
Frequency of hospitalization since diagnosed	
< 2 times	1 (0.8%)
2-5 times	83 (66.9%)
> 5 times	40 (32.3%)

(Source: Primary data, 2019)

### Data of SPICT indicators

The SPICT data based on general and clinical indicators are shown in Table 3. In the general indicators, most respondents answered the symptoms did not change even though they were getting optimal therapy which was followed by declining health conditions, dependence on others and some patients required hospitalization due to deteriorating physical conditions. Whereas, the clinical indicators showed decreasing in functional ability (cancer metastases), with physical conditions too weak to undergo treatment (cancer), some suffering from severe chronic lung disease, and many patients at risk of dying in less than 1 year.

**Table 3:** Percentage of NCD patients corresponding to general and clinical indicators (*n* = 124)

Indicators of SPICT	Indicators^a^	
	Yes (n/%)	No (n/%)
**General indicators (6 items)**^b^		
1. Unplanned hospital admission	68 (54.8%)	56 (45.2%)
2. Performance status is poor or deteriorating, with limited reversibility	84 (67.7%)	40 (32.3%)
3. It depends on others for care due to increasing physical and mental health problems.	76 (61.3%)	48 (38.7%)
4. The person has had significant weight loss over the last few months or remains underweight	48 (38.7%)	76 (61.3%)
5. Persistent symptoms despite optimal treatment of underlying conditions	109 (87.9%)	15 (12.1%)
6. The person (family) ask for palliative care; chooses to reduce, stop or not have treatment or wishes to focus on the quality of life	12 (9.7%)	112 (90.3%)
**Clinical Indicator (23 items)**^c^		
1. Too frail for cancer treatment or treatment is for symptom control	26 (21%)	98 (79%)
2. Functional ability deteriorating due to progressive cancer	52 (41.9%)	72 (58.1%)
3. Unable to dress, walk or eat without help	7 (5.6%)	117 (94.4%)
4. Eating and drinking less, difficulty with swallowing	4 (3.2%)	120 (96.8%)
5. urinary and faecal incontinence	0 (0%)	124 (100%)
6. Not able to communicate by speaking; little social interaction	2 (1.6%)	122 (98.4%)
7. Frequent falls; fractured femur	0 (0%)	124 (100%)
8. Recurrent febrile episode or infection; aspiration pneumonia	3 (2.4%)	121 (97.6%)
9. Progressive deterioration in physical and/or cognitive function despite optimal therapy	1 (0.8%)	123 (99.2%)
10. Speech problem with increasing difficulty communicating and/or progressive difficulty with swallowing	2 (1.6%)	122 (98.4%)
11. Recurrent aspiration pneumonia: breathless or respiratory failure	1 (0.8%)	123 (99.2%)
12. Persistent paralysis after stroke with significant loss of function and ongoing disability	1 (0.8%)	123 (99.2%)
13. Heart failure or extensive, untreatable coronary artery disease: with breathlessness or chest pain at rest or in minimal effort	5 (4.0%)	119 (96.0%)
14. Severe, inoperable peripheral vascular disease	4 (3.2%)	120 (96.8%)
15. Severe, chronic lung disease; with breathlessness at rest or on minimal effort between exacerbations	20 (16.1%)	104 (83.9%)
16. Persistent hypoxia needing long term oxygen therapy	4 (3.2%)	120 (96.8%)
17. Has needed ventilation for respiratory failure or ventilation is contraindicated	0 (0%)	124 (100%)
18. Stage 4 or 5 chronic kidney disease (eGFR < 30 ml/m) with deteriorating health	8 (6.5%)	116 (93.5)
19. Kidney failure complicating other life-limiting conditions or treatments	7 (5.6%)	117 (94.4%)
20. Stopping or not starting dialysis	2 (1.6%)	122 (98.4%)
21. Cirrhosis with one or more complications in the past year.	3 (2.4%)	121 (97.6)
22. Liver transplant is not possible	0 (0%)	124 (100%)
23. Deteriorating and at risk of dying with others conditions or complications that are not reversible; any treatment available will have a poor outcome	15 (12.1%)	109 (87.9%)

(Source: Primary data, 2019)

*SPICT* supportive and palliative care indicators tool

^a^indicators yes/no

^b^general indicators (6 items)

^c^clinical indicators (23 items)

### Identification of palliative care needs

In this study, the SPICT tool was used by the researchers to identify the need of palliative care of patients with NCD. Table 4 shows the distribution of palliative care needs of patients with NCD assessed by the researchers and clinicians (physicians and nurses). We found that based on the SPICT, 61% of the patients needed palliative care, while nurses thought this was the case for 18% of the patients and physicians for 10% of the patients. In addition, there was some dissimilarity in the assessments and perceptions between the clinicians (physicians and nurses) and researchers in assessing the palliative care needs. In a considerable number of cases, the clinician decided that a patient did not need palliative care, while the SPICT indicated that a patient needed palliative care (36% for nurses and 28% for physicians). The overall agreement of the clinician’s assessments to the researchers was < 32%; agreement with nurses was 32 and 25% with the physicians.

**Table 4:** Distribution of Palliative Care Needs on NCD patients using SPICT and based on screened by Physicians and Nurses. (*n* = 124)

Screening results	n (%)
Palliative screening by researcher using SPICT	
SPICT +	76 (61.3%)
SPICT -	48 (38.7%)
Palliative screening without SPICT by nurses	
Nurse +	22 (17.7%)
Nurse -	61 (49.2%)
Nurse Unidentified (difficult to identified)	41 (33.1%)
Palliative screening without SPICT by physician	
Physician +	12 (9.7%)
Physician -	80 (64.5%)
Physician Unidentified (difficult to identified)	32 (25.8%)
Similarity screening of nurse to researcher	
SPICT + /Nurse + and SPICT −/Nurse -	39 (31,5%)
SPICT + /Nurse -	45 (36,3%)
SPICT - / Nurse +	10 (8%)
SPICT +/− and Nurse unidentified	30 (24,2%)
Similarity screening of physician to researcher	
SPICT + / Physician + and SPICT−/Physician -	31 (25.0%)
SPICT + / Physician -	35 (28.2%)
SPICT. - / Physician +	29 (23,4%)
SPICT + /− and Physician unidentified	29 (23,4%)

(Source: Primary data, 2019)

### Number of NCD patients who need palliative care

In this study, 76 cases (61.3%) were categorized requiring palliative care based on the SPICT screening by the researchers. Patients required palliative care when the screening results show general indicators ≥2 and specific indicators ≥1. Table 5 shows that among patients with PC needs, the majority were female (59.2%) and their ages were < 60 years old (73.7%). Most of the respondents were suffering from cancer (67.1%) with a duration of illness of more than 6 months (92.2%). Table 5 also shows that there was significant difference in the length of time the patient suffered from the disease and the need for palliative care (*p* < 0.05), while sex, age, and type of disease did not have a significant difference in the need for palliative care.

**Table 5:** Palliative Care needs of NCD patients based on demographic characteristics (*n* = 124)

Characteristic	f (%)	General indicators		Clinical indicators		PC needs		
		Score ≥ 2 f (%)	Score ≤ 1 f (%)	Score ≥ 1 f (%)	Score 0 f (%)	Yes f (%)	No f (%)	*p*-Value
Sex								
Male	52 (41.9%)	46 (46.9%)	6 (23.1%)	32 (40.0%)	20 (45.4%)	31 (40.8%)	21 (43.7%)	0.745
Female	72 (58.1%)	52 (53.1%)	20 (76.9%)	48 (60.0%)	24 (54.6%)	45 (59.2%)	27 (56.3%)	
Age								
< 60 years	94 (75.8%)	74 (74.0%)	20 (83.3%)	53 (72.6%)	41 (80.3%)	56 (73.7%)	38 (79.2%)	0.487
≥ 60 years	30 (24.2%)	26 (26.0%)	4 (16.7%)	20 (27.4%)	10 (19.7%)	20 (26.3%)	10 (20.8%)	
Type of disease								
Cancer	89 (71.8%)	68 (68.0%)	21 (87.5%)	49 (67.1%)	40 (78.4%)	51 (67.1%)	38 (79.2%)	0.146
Non cancer	35 (28.2%)	32 (32.0%)	3 (12.5%)	24 (32.9%)	11 (21.6%)	25 (32.9%)	10 (20.8%)	
Duration of illness								
< 6 months	17 (13.7%)	10 (10.2%)	7 (26.9%)	7 (8.7%)	10 (22.7%)	6 (7.8%)	11 (22.9%)	0.018*
≥ 6 months	107 (86.3%)	88 (89.8%)	19 (73.1%)	73 (91.3%)	34 (77.3%)	70 (92.2%)	37 (77.1%)	

(Source: Primary data, 2019)

*f* frequency, *PC palliative care,* Indicator (general score ≥ 2, clinical score ≥ 1) means need PC

* significant < 0.05; CI 95%

## Discussion

The results highlight that more than 60% of hospitalized patients with NCDs have palliative care needs, which was often overlooked by clinicians. The results also showed there was high disagreement between clinicians in identifying the need of patients for palliative care. Therefore, an instrument could be helpful in detecting patients in need for palliative care.

Based on the distribution of NCD, the percentage of NCD requiring palliative care at Dr. Sardjito Hospital Yogyakarta was 61.3%. The high number of palliative cases indicate a high number of patients who need end of life care. These data are congruent with previous study that indicated around 80% of NCD become the primary cause of death in many hospitals in Yogyakarta [21, 22]. This situation illustrates to what extent NCD in Yogyakarta will become an even greater threat to health and life in the near future.

Early identification of NCD patients’ needs for palliative care can help patients to receive palliative care earlier which will have an impact on improving the patient’s quality of life [23, 24]. However, the differences in the judgments between physicians, nurses and researchers in screening the palliative care needs of patients with NCD represent a significant challenge in the early provision of palliative care. The study found that many clinicians are unaware of the need for palliative care for their patients. Several factors involving the knowledge and experience of clinicians regarding palliative care can influence these findings and influence their decisions in determining the screening process and referrals, for example:

1. The perceptions of referring patients for palliative care indicated that the clinicians are incapable to deal with the patients’ problems and/or have considered making less effort in treating the patient due to their end-of-life condition. Other physicians postponed the referring of patients to palliative care because they do not want to disappoint patients and their families, and/or they do not understand the benefits of referrals and see referrals as a recognition of their own failure [25]. Also, some believe that palliative care is not suitable for all patients [26]. This perception may be due to doctors who are considering the impact regarding the patients’ and family’s trust of the doctor, and thereby are delaying referrals to palliative care services [27].
2. The cost of medical services is based on medical treatment and not palliation. Based on the Republic of Indonesia Minister of Health Regulation number 27 of 2014 that stated health service tariffs at advanced health facilities and payment patterns are based on the provisions of the Indonesia Case Base Groups (INA-CBGs) which are using the current ICD-10 diagnosis codes [28]. In the Healthcare and Social Security Agency (BPJS) practical guide, it was mentioned the technical verification of claims is based on INA-CBGs [29]. However, in International Statistical Classification of Diseases and Related Health Problems (ICD)-10 there is no taxonomy of palliative diagnoses, so it is possible that claims against BJPS cannot be paid on a legal basis. Also, it can be said that palliative care has not yet been included in BPJS. This can be seen from the statement of the palliative team leader of Dr. Hasan Sadikin Hospital in Bandung who explained the present claims for palliative services have not been honored by BPJS [30]. Also, in the national guidelines for cancer palliative programs, the number of palliative service tariffs are determined based on treatment and drug tariffs, and the allocations of funding for palliative services are from the government and the private sector [31]. However, it is not mentioned in detail the specific amounts of this budgeting and their allocation.
3. The decision belongs to ‘the physician in charge’. There is the possibility of clinicians who have identified palliative conditions, but they must obey the existing rules. For the management of patients from admission to discharge, full responsibility rests on ‘the physician in charge’. All medical activities done to patients must be approved by ‘the physician in charge’ [32]. The management of inpatients in hospitals becomes the authority of ‘the physician in charge’ including diagnostic and therapeutic measures, referral, or discharge of patients so that their decision has legal jurisdiction. However, the challenges faced when there are obstacles/barriers to the presence of ‘physician in charge’ create implications for hospitalized patients and the process within the health care system. This dilemma has become a management concern related to the hospital policies in screening palliative patients, referrals and providing palliative care facilities [27].
4. There are inherent differences in the clinician’s knowledge in screening palliative patients. The doubtful response from the clinician in ascertaining the palliative condition indicates a variation in the ability to screen for palliative care cases. However, more in-depth research needs to be done to measure the level of professional knowledge about palliative care. Studies suggest that there are difficulties in the early detection of chronic disease cases that require further treatment [33]. A study found that only 30.4% of the nursing staff had knowledge of palliative care [34]. Nurses need to increase their knowledge because the palliative care domain requires more nursing roles. It is the essential aim of the nursing care principle to meet the needs of palliative patients [15].
5. Challenges and barriers to the provision of palliative care include the difficulty of having an open discussion with patients and families about the need for palliative care to patients. Therefore, clinicians need to be trained to be able to communicate and take a personal approach to the patient and family [33].

## Conclusions

This study found that more than half of the hospitalized patients with NCD required palliative care as identified by the researchers using the SPICT tool. These results also highlight that by using the SPICT tool, recognition of hospitalized patients with NCD needing palliative care increased from 10 to 18% to 63%. Prompt identification of palliative care needs of patients with NCD might improve the quality of palliative care and the patients’ quality of life. The Indonesian version of the SPICT tool can help the clinicians to reach agreement in identifying hospitalized patients who need palliative care as the first step in addressing palliative interventions for patients with NCD. Future qualitative studies are needed to elicit more detailed information on clinicians’ perceptions of the importance of identification of the need for palliative care in seriously ill patients.

## Data Availability

The datasets used and analyzed during the current study are not publicly available due to restraints of the ethical permit, some data may be available from the corresponding author upon reasonable request.

## Abbreviations

NCD: Non-communicable diseases
SPICT: Supportive & Palliative Care Indicators Tool (SPICT)
Kr: Coefficient of reproducibility validity
Ks: Coefficient of scalability validity
KR: Kuder-Richardson
INA-CBGs: Indonesia Case Base Groups
BPJS: Healthcare and Social Security Agency
ICD: International Statistical Classification of Diseases and Related Health Problems
DPJP: Clinician in charge
