# Correction to: Identifying palliative care needs of patients with non-communicable diseases in Indonesia using the SPICT tool: a descriptive cross-sectional study

**DOI:** 10.1186/s12904-022-00909-4

**Published:** 2022-02-05

**Authors:** Christantie Efendy, Jony Francisco Dos Santos Silva, Retna Siwi Padmawati

**Affiliations:** 1 Department of Medical Surgical Nursing, Faculty of Medicine, Public Health and Nursing, Universitas Gadjah Mada, Jl. Farmako, Sekip Utara, Depok District, Sleman, Special Region of Yogyakarta, Yogyakarta, 55281 Indonesia; 2 Center for Bioethics and Medical Humanities, Faculty of Medicine, Public Health, and Nursing, Universitas Gadjah Mada, Yogyakarta, Indonesia; 3 Hospital Nacional Guido Valadares, Dili, Timor-Leste; 4 Department of Health Behavior, Environment, and Social Medicine; Faculty of Medicine, Public Health, and Nursing, Universitas Gadjah Mada, Yogyakarta, Indonesia; 5 Graduate Study Program on Bioethics, Universitas Gadjah Mada, Yogyakarta, Indonesia

**Correction to: BMC Palliat Care 21, 13 (2022)**

**https://doi.org/10.1186/s12904-021-00881-5**

Following the publication of the original article [1], the last reference provided was not implemented during corrections stage. The last reference as below should have been included.

Jony Francisco Dos Santos Silva. Identifikasi Kebutuhan Palliative Care pada Pasien Penyakit Kronis di Ruang Rawat Inap Dewasa di RSUP Dr. Sardjito Yogyakarta. Thesis. 2020. Yogyakarta. Unpublished

The original article has been updated.
